# Division of Labor in the Hyperdiverse Ant Genus *Pheidole* Is Associated with Distinct Subcaste- and Age-Related Patterns of Worker Brain Organization

**DOI:** 10.1371/journal.pone.0031618

**Published:** 2012-02-17

**Authors:** Mario L. Muscedere, James F. A. Traniello

**Affiliations:** Department of Biology, Boston University, Boston, Massachusetts, United States of America; Stanford University, United States of America

## Abstract

The evolutionary success of ants and other social insects is considered to be intrinsically linked to division of labor among workers. The role of the brains of individual ants in generating division of labor, however, is poorly understood, as is the degree to which interspecific variation in worker social phenotypes is underscored by functional neurobiological differentiation. Here we demonstrate that dimorphic minor and major workers of different ages from three ecotypical species of the hyperdiverse ant genus *Pheidole* have distinct patterns of neuropil size variation. Brain subregions involved in sensory input (optic and antennal lobes), sensory integration, learning and memory (mushroom bodies), and motor functions (central body and subesophageal ganglion) vary significantly in relative size, reflecting differential investment in neuropils that likely regulate subcaste- and age-correlated task performance. Worker groups differ in brain size and display patterns of altered isometric and allometric subregion scaling that affect brain architecture independently of brain size variation. In particular, mushroom body size was positively correlated with task plasticity in the context of both age- and subcaste-related polyethism, providing strong, novel support that greater investment in this neuropil increases behavioral flexibility. Our findings reveal striking levels of developmental plasticity and evolutionary flexibility in *Pheidole* worker neuroanatomy, supporting the hypothesis that mosaic alterations of brain composition contribute to adaptive colony structure and interspecific variation in social organization.

## Introduction

Comparing neuroanatomical variation among individuals within and between invertebrate and vertebrate species has proven fruitful for exploring links between brain, behavior, ecology, life history and evolution [1]. Studies typically derive from the fundamental axiom that neural tissue is energetically expensive and thus relative investment in functionally distinct brain areas is shaped by natural selection on the sensory capabilities and cognitive behaviors supported by these regions [2]. Analyses have correlated brain structure and organization with diet, olfactory and visual acuity, group structure, and cognitive traits such as memory capacity in taxa as varied as birds, mammals and insects, providing important insights into the forces shaping animal nervous systems [3]–[18]. Sociality has received particular attention as a selective agent potentially favoring the evolution of larger brains and more elaborate higher-level integration regions [3].

**Figure 1:**
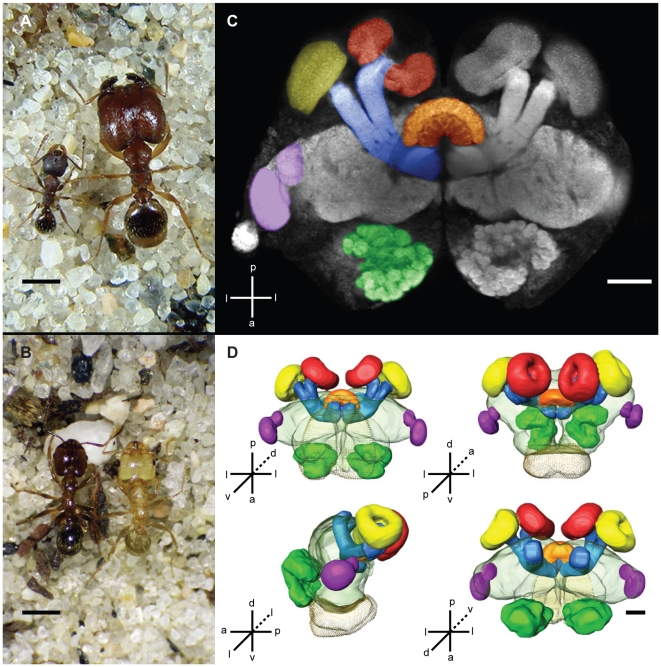
*Pheidole* worker morphology and neuroanatomy. (**A**) Mature *P. pilifera* minor (left) and major (right) workers. (**B**) *P. dentata* mature (left) and young (right) major workers, identifiable by cuticle colour. (**C**) Confocal micrograph of a *Pheidole* worker brain in horizontal section showing regions of neuropil. Central brain subregions visible in this section are pseudocolored. Section is slightly oblique, only one optic lobe is visible in the plane of sectioning. (**D**) 3D reconstructions of the brain shown in (**C**). Central brain functional subregions are rendered solidly, other central brain neuropils are transparent grey, and the subesophageal ganglion is a point cloud. Key: a, anterior; p, posterior; d, dorsal; v, ventral; l, lateral; green, antennal lobe; orange, central body; red, mushroom body medial calyx; yellow, mushroom body lateral calyx; blue, mushroom body peduncle & lobes; purple, optic lobe. Scale bars are 1 mm in (**A**) and (**B**) and 50 µm in (**C**) and (**D**).

Ants are exemplars of social life, and their extraordinary diversity and abundance is frequently attributed to the division of labor and collective behavior that underscore colony operations [19]. Although the question of how miniaturized ant brains generate adaptive behavior was posed at least as early as the 19^th^ century, when Darwin mused on the “extraordinary mental activity” of such an “extremely small absolute mass of nervous matter” [20], explorations of the association of sociality, ecology and neuroanatomical variation in ants and other social insects are still nascent [17], [18], [21]–[24]. The hyperdiverse ant genus *Pheidole* offers an ideal system in which to investigate these relationships because of its remarkable adaptive radiation, striking degree of intra- and interspecific variation in worker behavior, and strong subcaste morphological differentiation and division of labor, which is considered key to the diversification of the genus [25]. As in many eusocial hymenopterans, *Pheidole* workers develop behaviorally and change task performance patterns as they age: only older individuals work outside the nest, where they encounter a more complex and heterogeneous sensory environment than younger workers residing within dark nest chambers below ground or within wood. In *Pheidole dentata*, minor workers undergo behavioral maturation during the first few weeks of adult life by adding outside-nest tasks such as foraging to their existing within-nest activities [26]. During this maturational period, *P. dentata* minors become more efficient at some tasks [27] and their cephalic musculature concurrently matures [28]. Additionally, their brains experience synaptic remodeling [29], changes in biogenic amine content [30], and increased levels of serotonergic immunoreactivity [31]. Division of labor in *Pheidole* based on morphology involves subdivision of workers into discrete physical subcastes (Figure 1A), which often have distinct behavioral roles. More numerous minor workers attend to a broad range of tasks including brood care, nest maintenance, and foraging, while major workers (“soldiers”), which are larger in size and have disproportionately large heads, typically specialize on colony defense and seed milling [25], compromising their task plasticity [32]. Lastly, there is considerable variation among the ca. 1,100 *Pheidole* species in colony size, nesting ecology, diet, body size, subcaste investment, and in behavioral traits such as foraging mode, worker aggressiveness, and the breadth and plasticity of major worker behavior [25], [32], [33].

Is the diversity of division of labor among *Pheidole* workers of different species associated with variation in brain structure? Investigations of the neuroanatomical correlates of insect behavior often focus solely on the mushroom bodies (MBs), a sensory integration and processing region of the brain considered vital for learning, memory, and behavioral flexibility [24], [34]–[37]. Evidence supporting the role of the MBs as neural substrates important for behavioral flexibility in social hymenopterans includes correlations between age-related changes in task performance and MB development. Older workers generally have larger MBs than younger workers, presumably reflecting the greater sensory processing and behavioral plasticity required for efficient outside-nest work [38]–[41]. However, chronological age and prior task experience, when experimentally uncoupled [38], [40], [42]–[44], both affect MB size. MB growth in adult social hymenopterans thus likely reflects both “experience-expectant” canalized developmental processes that precede worker behavioral transitions and “experience-dependant” plastic responses to task performance [43], [45], [46]. MB size also tracks reproductive division of labor: the MBs of behaviorally flexible worker ants are relatively larger compared with the MBs of conspecific sexuals specialized on reproduction [47]. The volume of the MBs increases in locusts when they transition from solitary to gregarious phases, which are associated with increased dietary generalism [7]. Comparative studies arguing that structurally complex MBs have evolved due to the cognitive demands of a generalist diet in coleopterans [10], and parasitoid life history in hymenopterans [11] further support a general coupling of behavioral plasticity and MB size in insects. While studied less frequently, age- and task-related size variation has also been documented in the antennal lobes and other neuropil compartments in social insects [17], [18], [40]. Worker behavioral differentiation, therefore, is probably not generated solely by differences in MB size or organization, but by adaptive patterns of brain organization that reflect the integrated functional roles of all brain subregions in task performance. Studies of the relative investment by workers in multiple functionally distinct brain subregions – which globally can be considered a worker's neural phenotype – may thus provide a more holistic and detailed understanding of the interplay between behavior, ecology, evolution, sociality, and neuroanatomy.

Here we provide the first comprehensive scaling analysis of multiple, functional brain subregions and the first description of the associations between brain structure, age, and body size in a social insect. Using multivariate methods and a robust sample of *Pheidole* brains collected from workers that vary in age, subcaste, and species, we first determined if brain organization differed among behaviorally differentiated worker groups. We predicted that selection imposed by variation in species ecology and social environments of workers of different subcaste and age favored distinctive patterns of brain structure. Second, we analyzed how individual brain subregions scaled with brain size; we predicted differences in neural phenotype among worker groups could not be explained solely by straightforward developmental scaling relationships, but would illustrate adaptive patterns of interspecific variation and intraspecific developmental plasticity. Finally, we determined whether variation in brain structure was correlated with behavioral differences among worker subcaste and age groups in our focal species. We predicted that plasticity in the sizes of sensory input (optic and antennal lobes) and processing structures (MBs) would be associated with species-typical patterns of division of labor. Examining this prediction integrated the analysis of two classical correlates of division of labor – age and morphology – with neuroethological concepts of task plasticity that suggest variation in behavioral flexibility and specialization among workers are the result of differential elaboration of sensory input and processing regions, including the MBs.

## Materials and Methods

### Ethics statement

Colonies were maintained in the lab in compliance with conditions specified in USDA APHIS PPQ permit number P526-11-01197, and earlier issues of the permit. Culture methods maximized colony growth and health. No specific permits were required to collect colonies in the field and none of our focal species are endangered or protected. Colonies were collected from unprotected public land with no measurable habitat disturbance.

### Species selection, ecology and natural history of focal species, and ant husbandry

We selected three North American *Pheidole* species (*P. dentata*, *P. morrisi* and *P. pilifera*) whose sociobiology, ecology, and life history encompass the broad variation in colony structure and division of labor identified in this genus [25]. The most salient interspecific differences concern colony size (*P. morrisi*>*P. dentata*>*P. pilifera*), diet (*P. morrisi* and *P. dentata* are scavengers, *P. pilifera* is primarily granivorous), and major worker investment and behavioral plasticity (*P. morrisi*>*P. dentata*>*P. pilifera*). *Pheidole dentata* is a small-bodied, ground- and wood-nesting ant species native to the mid-Atlantic to southeastern United States and west to Illinois, Texas and northern Mexico. Colonies often contain more than 1,000 workers and forage on live and dead insect prey. Typically, majors are uninvolved in most colony tasks, although they leave the nest to provide resource and colony security, including enemy-specific defensive behavior [48]. *Pheidole morrisi* is distributed along the east coast of the U.S. from Florida to Long Island, New York. *Pheidole morrisi* colonies are also insectivorous, ground-nesting scavengers, but are larger (often >10,000 workers) and competitively dominant, with numerous foraging workers that aggressively defend the nest and compete with sympatric ant species. Colonies contain a relatively high proportion of major workers that are unusually active in some tasks typical of *Pheidole* minors [49], including foraging and other outside-nest work. *Pheidole pilifera* is widely distributed throughout the continental United States. Colonies nest in the ground, are comparatively small (∼600–800 workers), and characterized by relatively small numbers of “shy” foragers that either flee or feign death in response to danger. *Pheidole pilifera* colonies have a low proportion of large, docile majors that rarely leave the nest and have a repertoire restricted largely to seed milling and blocking nest passages. *Pheidole pilifera* colonies scavenge insect prey but predominantly collect, store, and feed on seeds. Major workers are typically found in nest chambers where seeds are cached.

Queenright colonies were collected in Centereach, NY (*P. pilifera* and *P. morrisi*), Rocky Point, NY (*P. morrisi*), Concord, MA (*P. pilifera*), and Alachua County, FL (*P. dentata*), and maintained in the laboratory using established protocols [26], [27]. Newly eclosed callows (0–2 days old) and fully mature workers from all three species were identified by cuticular coloration [26], [27], [29]–[31]. Callow workers in all three species have uniform light yellow cuticles that darken predictably over the first few weeks of adult life (Figure 1B), a process that takes 16–20 days in *P. dentata* minor workers.

### Immunohistochemistry and confocal microscopy

Individual worker brains were dissected from the head capsule in cold insect Ringer's solution, quickly transferred to cold Dent's fixative (4∶1 absolute methanol∶dimethyl sulfoxide [DMSO]), and fixed for 12 hours at −20°C (with one change into fresh fixative after ∼30 min). Brains were washed 1 time in absolute methanol, then stored in absolute methanol at −20°C until further processed. Brains were rehydrated through a graded methanol series (95%, 70%, 50%, 30%, PBS, for 5 min each), washed (6×10 min) in 0.01 M PBS with 1% bovine serum albumin and 1% DMSO (PAD), incubated for 1 hour in a blocking solution of 10% normal goat serum in PAD (PADN), and incubated for 60 hours at room temperature in the primary antiserum SYNORF1, diluted 1∶50 in PADN. We initially obtained SYNORF1 as a gift from Dr. E. Buchner, University of Würzburg; SYNORF1 was later obtained from the Developmental Studies Hybridoma Bank (developed under the auspices of the NICHD and maintained by The University of Iowa, Department of Biology, Iowa City, IA 52242). After incubation, brains were washed (6×10 min) in PAD and incubated in the dark for 36 hours in a solution of Alexa Fluor 568 goat anti-mouse secondary antibody diluted 1∶200 in PADN. Stained brains were washed (3×10 min) in PAD, washed (3×10 min) in 0.01 M PBS, and dehydrated through a graded methanol series (30%, 50%, 70%, 95%, 100%, 100%, for 5 min each). Dehydrated brains were transferred to a 30% methyl salicylate/70% methanol solution for 5 min, then transferred to a dish of 100% methyl salicylate and allowed to fully clear. Cleared brains were whole-mounted in 100% methyl salicylate using custom-made double-sided steel well slides.

Brains were imaged using an Olympus Fluoview BX50 laser-scanning confocal microscope with a 20× objective (N.A. = 0.5). Labeled structures were excited with a Krypton laser at 568 nm and their fluorescence detected using a 605/45 nm band-pass filter. Brains were optically sectioned in their entirety through the horizontal plane at 0.6 µm increments. To correct for axial shortening along the z-axis introduced by the refractive index mismatch between air and methyl salicylate [50], we multiplied the nominal section thickness (0.6 µm) by a correction factor of 1.59 to obtain the true section thickness of approximately 1 µm (0.954 µm). We stained and imaged 10 brains from each of 12 worker groups (young minors, young majors, mature minors, and mature majors from *P. morrisi*, *P. pilifera*, and *P. dentata*), for a total *n* = 120 worker brains.

### Neuroanatomical measurements

We used Amira v3.1 and ImageJ v1.41d to measure the volume of one neuropil hemisphere and seven of its functional subregions from each brain (Figure 1C,D): the lobula and medulla of the optic lobe (OL), the antennal lobe (AL), the mushroom body (MB) medial calyx, the MB lateral calyx (both calyces together denoted as MB-C), the MB peduncle, including the vertical and medial lobes (MB-PL), the central body (CB), and the subesophageal ganglion (SEG). Because the SEG neuropil is fused and completely contiguous with the central brain (supraesophageal ganglion) neuropil in *Pheidole*, we delineated this structure from the central brain by assigning all neuropil ventral to the esophageal foramen to the SEG. To capture shape variation among brains in addition to volume differences, we made six more linear measurements in horizontal section where well-defined anatomical landmarks could be identified: MB medial calyx maximal width, MB lateral calyx maximal width, MB peduncle maximal width at its point of entry into the protocerebral lobe, CB maximal height and width, and SEG width.

### Statistical analyses

Multivariate discriminant analyses were conducted to assess overall variation in neural phenotype among groups. All size and shape variables were size-corrected and included in these analyses (as central brain subregion volume/remainder-of-central brain volume [ROCBV, calculated separately for each brain subregion], SEG volume/central brain volume [CBV], or linear shape measurement/maximal width of central brain neuropil across the protocerebrum). Scaling between individual brain subregions and brain size was analyzed using standardized major axis (SMA) regression with the program (S)MATR v. 2.0 [51]. The contributions of between-groups effects (i.e., effects of age or subcaste) to scaling differences identified with (S)MATR were assessed with traditional ANCOVA. Finally, we tested for differences among worker groups in the absolute volumes of the OLs, ALs, and MBs, and for differences in relative brain size among mature worker groups, using ANOVA. Full statistical details are presented as Supporting Information S1.

## Results and Discussion

### *Pheidole* worker subcastes, age groups, and species have distinct brain architectures

**Figure 2:**
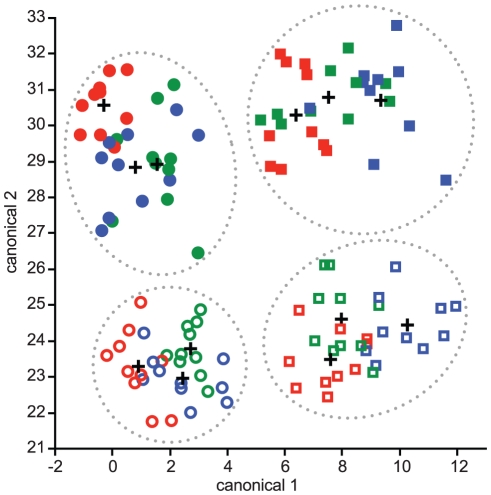
*Pheidole* subcastes and age groups have neural phenotypes that are well separated in multivariate space. Plot shows the separation of all 12 worker groups on the first two canonical variables generated by discriminant analysis (accounting for 87% of the total variance explained by the analysis). Four non-overlapping clusters (dotted ellipses) are generated by very strong discrimination among young minors, old minors, young majors, and old majors. Key: red, *P. dentata*; green, *P. morrisi*; blue, *P. pilifera*; open, young age cohort; closed, mature age cohort; circles, minor workers; squares, major workers; crosses, group centroids.

**Figure 3:**
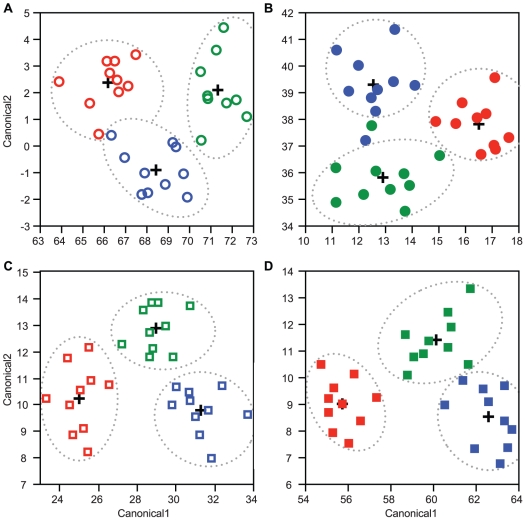
Interspecific variation in *Pheidole* multivariate neural phenotypes. Separate discriminant function analyses reveal significant differentiation of neural phenotype among species for (**A**) young minors, (**B**) old minors, (**C**) young majors, and (**D**) old majors. Key: red, *P. dentata*; green, *P. morrisi*; blue, *P. pilifera*; open, young age cohort; closed, mature age cohort; circles, minor workers; squares, major workers; crosses, group centroids.

Multivariate discriminant analysis revealed highly significant gross neuroanatomical differentiation among our 12 worker groups (Wilks' *Λ* = 4×10^−4^, *p*<0.0001). The model precisely classified brains to correct species, age cohort, and subcaste in 92.5% of cases (Figure 2), significantly exceeding chance expectations (8.3%; Press's *Q* = 1.11×10^−4^, *p*<0.0001). All incorrectly classified brains were correctly identified to age group and subcaste but not species. Reanalysis using only age cohort and subcaste as the four *a priori* groups enhanced discrimination, correctly classifying all 120 brains (Wilks' *Λ* = 8.92×10^−3^, *p*<0.0001; 100% correct, *Q* = 360, *p*<0.0001). However, separate discriminant analyses of data subsets using only species identity as *a priori* categories confirmed significant interspecific differentiation (Table 1, Figure 3) for young minors (Wilks' *Λ* = 0.049, *p* = 0.0002), mature minors (Wilks' *Λ* = 0.067, *p* = 0.0010), young majors (Wilks' *Λ* = 0.039, *p*<0.0001), and mature majors (Wilks' *Λ* = 0.037, *p*<0.0001). More conservative jackknifed (leave-one-out) classification designs, in which each brain was categorized by discriminant functions derived using only the other *n*-1 samples, also had high correct classification rates that significantly exceeded chance levels in all cases (Table 1). When using only age cohort and species as *a priori* groups, jackknifed classification was nearly as accurate as whole-model classification (99.2% correct), emphasizing that worker age cohorts and subcastes have strikingly differentiated neural phenotypes (Table 1).

**Table 1:** Results of discriminant function classification of worker brains to the *a priori* groups listed.

data subset	*a priori* groups (*n*)	classification scheme	correct classifications	*Q*	*p*
full dataset	species, age, subcaste (12)	full	92.5%	1.11×10^−4^	*<0.0001*
		jackknifed	79.2%	788	*<0.0001*
full dataset	age, subcaste (4)	full	100%	360	*<0.0001*
		jackknifed	99.2%	352	*<0.0001*
young minors	species (3)	full	96.7	54.2	*<0.0001*
		jackknifed	63.3%	12.2	*0.0005*
mature minors	species (3)	full	90%	43.4	*<0.0001*
		jackknifed	70.0%	18.2	*<0.0001*
young majors	species (3)	full	100%	60.0	*<0.0001*
		jackknifed	76.7%	25.4	*<0.0001*
mature majors	species (3)	full	100%	60.0	*<0.0001*
		jackknifed	73.3%	21.6	*<0.0001*
young workers	species, subcaste (6)	full	100%	300	*<0.0001*
		jackknifed	80.0%	173	*<0.0001*
mature workers	species, subcaste (6)	full	91.7%	243	*<0.0001*
		jackknifed	80.0%	173	*<0.0001*

Each row indicates classification outcomes using either the full discriminant model or the jacknifed (leave-one-out) model for the given analysis. Significant Press' *Q* statistics indicate that workers in a given analysis were classified to the correct *a priori* group at greater-than-chance levels.

Task experience can lead to neuroanatomical changes; differences among worker age cohorts in our analysis could therefore reflect experience-dependent and/or independent effects on brain development [38], [40], [42]–[46]. We tested whether discrimination among subcastes and species was enhanced among mature individuals, which have more diverse task repertories and greater task experience than recently eclosed workers, and thus greater potential for experience-related divergence in neural phenotype. Resulting whole-model classification of worker brains to subcaste and species was in fact slightly more accurate in young workers (Wilks' *Λ* = 0.0035, *p*<0.0001; 100% correct, *Q* = 300, *p*<0.0001) than in mature workers (Wilks' *Λ* = 0.0052, *p*<0.0001; 91.7% correct, *Q* = 243, *p*<0.0001), and the accuracy of jackknifed classification was equivalent for both age cohorts (80%, Table 1). This suggests that much of the neuroanatomical differentiation among subcastes and species is generated by alternative developmental scaling of brain regions prior to adult emergence, notwithstanding additional plastic responses to worker experience during adult life.

### Differentiated brains result from mosaic alterations of subregion scaling relationships

**Figure 4:**
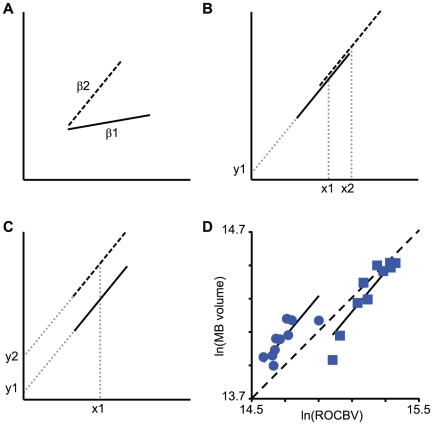
Examples of altered brain scaling relationships. Solid and dashed lines in (**A**–**C**) indicate hypothetical standardized major axis (SMA) scaling relationships of brain region volume (y variable) with brain size (x variable). (**A**) Altered slopes (β1 and β2), which preclude further scaling analyses using these methods. (**A**). Groups share a common scaling slope and y-intercept (y1) but average brain size differs between groups (×1 and ×2), producing an x-axis shift. (**C**) Groups share a common scaling slope and average brain size (×1) but y-intercepts (y1 and y2) are shifted, producing a grade shift. (**D**) Solid and dashed lines represent actual SMA scaling regressions of mushroom body volume versus remainder-of-central-brain volume (ROCBV) for mature *P. pilifera* minors (circles) and mature *P. pilifera* majors (squares), representative of a grade shift accompanying a shift along the x-axis. Dashed line in (*d*) indicates slope of 1 (isometry) for reference.

We conducted bivariate scaling analyses [51] of individual brain subregions to explore differences in brain composition that could lead to the highly differentiated brains identified by our discriminant models. These analyses revealed scaling relationships among brain subregions and total brain size that included differences among *Pheidole* worker groups in log-log slope, y-intercept, and position of scaling relationships along the x-axis (Figure 4, Table 2). Slopes (β) differed significantly among worker groups (Figure 4A) for the scaling of OL volume (*χ* ^2^ = 22.3, d.f. = 11, *p* = 0.02) and CB volume (*χ* ^2^ = 21.4, d.f. = 11, *p* = 0.03) with brain size, precluding further analyses. For scaling of other substructures with brain size, slopes did not significantly differ among groups. These relationships were characterized by different levels of nonlinearity, from isometry between SEG volume and CBV (β = 0.99) to positive allometry between MB-C volume and ROCBV (β = 1.25, Table 2). Allometric scaling of brain subregions could lead to the altered brain proportions among worker groups identified by our discriminant analyses simply because the groups differed significantly in central brain volume (ANOVA: *F*_11,108_ = 20.5, *p*<0.0001), resulting in significant shifts in the position of worker groups along the x-axis (Figure 4B) for all scaling comparisons with common slopes (Table 2 column 6). Nevertheless, these scaling relationships all exhibited significant grade shifts (different y-intercepts, Figure 4*c*) among worker groups (Table 2 column 5, Table 3, Table S1), which had strong effects on brain proportions independent of brain size differences. Grade shifts are particularly meaningful in brain scaling comparisons (e.g. [7], [52]) because they describe cases in which a structure is systematically larger or smaller in one group, and thus clearly indicate differential patterns of neural investment unrelated to differences in total brain size.

**Table 2:** Brain subregions scale differently with brain size across *Pheidole* worker groups.

y	x	scaling slope (β)	β different from 1?	grade shifts?	x-axis shifts?
SEG volume	CBV	0.99	no	yes	yes
		(0.89, 1.10)	(*χ* ^2^ = 0.075,	(*W* ^2^ = 324,	(*W* ^2^ = 405,
			*p* = 0.8)	*p<0.0001*)	*p<0.0001*)
AL volume	ROCBV	0.97	no	yes	yes
		(0.88, 1.09)	(*χ* ^2^ = 0.305,	(*W* ^2^ = 137	(*W* ^2^ = 227,
			*p* = 0.6)	*p<0.0001*)	*p<0.0001*)
MB volume	ROCBV	1.20	yes	yes	yes
		(1.10, 1.33)	(*χ* ^2^ = 14.8,	(*W* ^2^ = 518,	(*W* ^2^ = 220,
			*p<0.0001*)	*p<0.0001*)	*p<0.0001*)
MB-C volume	ROCBV	1.25	yes	yes	yes
		(1.15, 1.36)	(*χ* ^2^ = 23.5,	(*W* ^2^ = 267,	(*W* ^2^ = 188,
			*p<0.0001*)	*p<0.0001*)	*p<0.0001*)
MB-PL volume	ROCBV	1.15	yes	yes	yes
		(1.04, 1.29)	(*χ* ^2^ = 6.71,	(*W* ^2^ = 711,	(*W* ^2^ = 287,
			*p* = *0.01*)	*p<0.0001*)	*p<0.0001*)
CB volume	ROCBV	worker groups differ	yes for two groups	n/a	n/a
OL volume	ROCBV	worker groups differ	yes for five groups	n/a	n/a

We tested whether worker groups shared common scaling slopes, whether there were deviations from isometric scaling (β = 1), and whether there were significant shifts among worker groups in scaling elevation (grade shifts) or position along the x-axis (Figure 4, [51]). Values in parentheses indicate 95% confidence intervals of slopes (column 3) or statistical details (*χ* ^2^-tests, column 4; Wald tests, columns 5 and 6). All variables ln-transformed before analysis. CBV, central brain volume; ROCBV, remainder-of-central-brain volume.

### Intraspecfic variation in sensory neuropil investment correlates with worker task performance

We interpreted the ubiquity and magnitude of grade shifts in our dataset as evidence that the distinct patterns of neuropil investment of *Pheidole* worker groups are a functionally relevant outcome of adaptive developmental processes, rather than a by-product of scaling constraints among brains of different sizes. We therefore predicted that neural phenotypes should underscore variation in task performance among worker groups. Within *Pheidole* colonies, behavioral differences primarily exist along two non-mutually exclusive axes defined by subcaste (minors vs. majors) and age (mature vs. young). In both cases, groups working more frequently outside the nest (mature workers, minors) are faced with challenging navigational and sensory discrimination tasks, and have a larger behavioral repertoire than other groups that leave the nest rarely if ever (young minors) or less frequently (majors). We thus predicted that investment in neuropils that receive and integrate sensory stimuli (the OL, AL, and MBs), which are likely critical for worker task performance, would vary in similar ways along the subcaste and age axes. Patterns of investment fit this prediction (Table 4). Controlling for all other variables, mature workers (relative to young workers) and minors (relative to majors) had proportionally larger ALs and MBs (Table 3). OL scaling could not be analyzed using ANCOVA to hold all other variables constant because slopes differed among worker groups. However, averaging across all worker groups, OL differences also conformed to this prediction, being proportionally smaller in mature workers and minors (young vs. old: *t*_118_ = 3.71, *p* = 0.0003, 2.9±0.08% vs. 2.5±0.07%; major vs. minor: *t*_118_ = 10.0, *p*<0.0001, 3.1±0.07% vs. 2.2±0.05%).

**Table 3:** Between-group grade shifts in *Pheidole* brain subregion scaling are indicated by significant ANCOVA effects.

brain region	effect	*p*	description of grade shift (at equivalent brain size)
SEG	age	*<0.0139*	larger in mature workers
	species×subcaste	*<0.0089*	larger in majors; subcaste difference is greater in *P. pilifera*
AL	age	*<0.0001*	larger in mature workers
	species×subcaste	*<0.0002*	larger in minors; subcaste difference is greatest in *P. dentata*, smallest in *P. morrisi*
MB	species	*<0.0043*	largest in *P. dentata*, smallest in *P. pilifera*.
	age	*<0.0001*	larger in mature workers
	subcaste	*<0.0001*	larger in minors
MB-C	subcaste	*<0.0001*	larger in minors
	species×age	*<0.0247*	larger in mature workers; age difference is greatest in *P. pilifera*, smallest in *P. dentata*
MB-PL	species×subcaste	*<0.0289*	larger in minors; subcaste difference is smallest in *P. morrisi*
	species×age	*<0.0094*	larger in mature workers; age difference reduced in *P. morrisi*

Factor main effects are not listed for significant interaction terms. Grade shift descriptions are based on inspection of marginal means. Full ANCOVA model details are given in Table S1.

**Table 4:** Patterns of behavioral and sensory neuropil variation are concordant between worker age cohorts and subcastes.

		outside-nest	brain sensory subregions			brain motor subregions	
worker groups	behavioral repertoire size	task frequency	relative MB size	relative AL size	relative OL size	relative CB size	relative SEG size
age cohorts	mature>young	mature>young	mature>young	mature>young	mature<young	mature<young	mature>young
subcastes	minor>major	minor>major	minor>major	minor>major	minor<major	minor = major	minor<major
*concordance?*	*yes*	*yes*	*yes*	*yes*	*yes*	*no*	*no*

These patterns are consistent with a central role of olfaction in generating behavioral flexibility in *Pheidole*. Larger olfactory input regions (AL glomeruli) are associated with increased olfactory sensitivity [18], [53], and comparatively large MBs are associated with behavioral flexibility and heterogeneous sensory environments [7], [10], [11] in several taxa. In social hymenopterans, MB and AL growth in adults is correlated with age-related behavioral maturation [38], [40], [41], [54]. Our results agree with these findings and extend these analyses beyond age-related development to behaviorally differentiated worker subcastes. The fact that *Pheidole* minors have relatively larger MBs and ALs than majors supports the hypothesis that the size of these neuropils is related to worker task diversities and proficiencies and validates the linkage of MB size and behavioral plasticity in insects.

Olfaction is the dominant sensory modality of *Pheidole*; workers have small eyes with few facets (minors: ca. 50 ommatidia per eye; majors: ca. 75 ommatidia per eye), and foraging, defense, and colony emigration are organized predominantly by pheromones [19]. It is therefore not surprising that worker OLs are very small (grand mean across all worker groups: 2.7±0.06% of central brain volume), reflecting the limited role of vision in task attendance [24]. Smaller proportional OL sizes in workers more likely to perform tasks outside the nest (mature workers and minors), while seemingly counterintuitive, may simply reflect a disproportionate investment in odor-processing neuropils rather than a reduction in visual ability *per se*. In support of this interpretation, absolute OL volumes did not significantly differ between age cohorts (contrasts of least-square means from factorial ANOVA [Table S2]: minors, *F*_1,112_ = 3.04, *p* = 0.08, 1.01±0.05×10^−4^ vs. 8.77±0.05×10^−5^ mm^3^ for young vs. mature workers, respectively; majors, *F*_1,112_ = 3.81, *p* = 0.054, 1.62±0.05×10^−4^ vs. 1.77±0.05×10^−4^ mm^3^), while other neuropil regions including the ALs (minors, *F*_1,112_ = 5.75, *p* = 0.0181, 5.40±0.2×10^−4^ vs. 5.94±0.2×10^−4^ mm^3^; majors, *F*_1,112_ = 77.0, *p*<0.0001, 5.51±0.2×10^−4^ vs. 7.47±0.2×10^−4^ mm^3^) and MBs (minors, *F*_1,114_ = 14.9, *p* = 0.0002, 1.25±0.04×10^−3^ vs. 1.49±0.04×10^−3^ mm^3^; majors, *F*_1,114_ = 101, *p*<0.0001, 1.23±0.04×10^−3^ vs. 1.86±0.04×10^−3^ mm^3^) grew significantly larger in absolute volume in mature workers. Thus for age cohorts, differences in relative OL size seem to be associated largely with the growth of olfactory processing brain regions, which consequently alter the proportional sizes of other brain neuropils including the OLs. Similarly, differences in OL size between worker subcastes are likely due in large part to differences in eye size caused by the allometric scaling of head size that generates dimorphic subcastes in *Pheidole*.

Motor control subregions of the *Pheidole* central nervous system, which would not be predicted to affect worker behavioral flexibility in the same way as sensory input and processing regions, did not vary similarly in size along the subcaste and age axes (Table 4). As with the OL, CB scaling could not be analyzed with ANCOVA due to heterogeneity of scaling slopes among worker groups. Across all worker groups, the CB was relatively larger on average in young workers, but did not differ in relative size among subcastes (young vs. old: *t*_118_ = 7.89, *p*<0.0001, 0.86±0.01% vs. 0.71±0.01%; major vs. minor: *t*_118_ = 0.3, *p* = 0.76, 0.78±0.02% vs. 0.77±0.02%). As CB function is not well characterized in any ant, the consequences of variation in CB size are speculative. Differences in SEG volume relative to CBV were also dissimilar along the young-mature and major-minor axes (Table 4): the SEG was proportionally larger in mature workers and in majors (Table 3). The SEG is associated with the motor control of the mandibles, the principal tools used by ants to work, and with the control of other mouthparts as well as integration of their afferent sensory information. The mandibular muscles occupy most of the head capsule in *Pheidole* workers and undergo significant growth after eclosion as *P. dentata* workers age [28]. The same may be true in other species. Furthermore, majors of all three species, which have enlarged heads and mandibular muscles relative to conspecific minors, had much larger SEGs relative to CBV (Table 3). This pattern mirrors that shown for several species of termites with defensive biting soldiers, which have larger SEGs than conspecific pseudergates (“workers”) [55]. Together, these results suggest differences in relative SEG size among worker groups may at least in part represent underlying relationships between mandibular size, musculature, and sensory/motor innervation of these structures.

### Interspecific variation in subcaste brain organization correlates with life history

**Figure 5:**
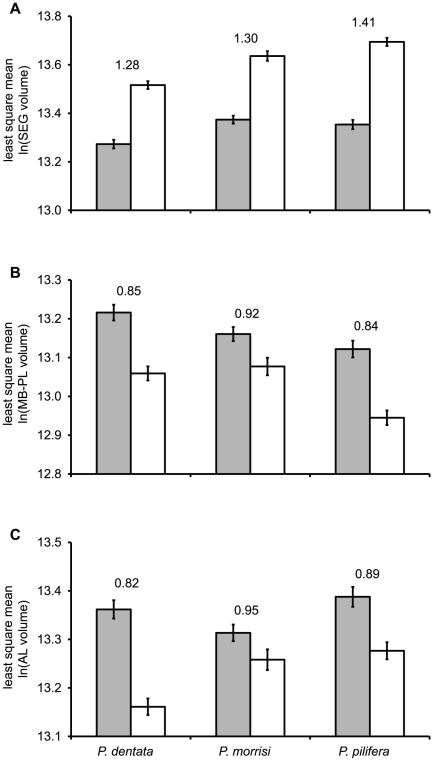
Interspecific variation in subcaste brain structure scaling. Least-square mean (± s. e. m.) brain subregion sizes from ANCOVA model (Table 3, Table S1) illustrating significant species×subcaste interactions for ln-transformed (**A**) subesophageal ganglion volume, (**B**) mushroom body peduncle and lobes volume, and (**C**) antennal lobe volume. Numbers above pairs of bars are major-to-minor-worker ratios of (untransformed) least-square means for each species. Key: grey bars, minor workers; open bars, major workers.

The three taxa chosen characterize a significant range of variation in behavioral and morphological divergence among *Pheidole* species and subcastes. We therefore examined whether the significant species×subcaste interactions in our ANCOVA analysis (SEG, AL, and MB-PL, Table 3) revealed relationships between the degree of major worker specialization and species life history. Majors of *P. dentata* and *P. morrisi* had SEGs approximately 28–30% larger, controlling for CBV and worker age, than conspecific minor workers (Figure 5A). While the degree of morphological difference among majors and minors is similar in *P. dentata* and *P. morrisi*, *P. pilifera* majors are proportionally much larger than their conspecific minors and have large heads with blunt mandibles used to grind seeds. In *P. pilifera*, SEGs were approximately 41% larger in majors than in minors after controlling for central brain volume and worker age. Although it is unclear whether or how SEG size scales with the size and/or closing power of the mandibular musculature, enlarged SEGs in *P. pilifera* majors could reflect sensory/motor specializations for the use of their mouthparts. Significant species×subcaste interactions were also evident for the ALs and one component of the MBs, the pedunculi and lobes (MB-PL). Of our three focal species, *P. morrisi* minors and majors are notable for their convergence in behavioral diversity: majors are active and engage in many of the same tasks as conspecific minors, including foraging [25], [49]. After correcting for ROCBV and worker age, the differences in MB-PL and AL sizes between subcastes were smallest in *P. morrisi* (Figure 5B,C), mirroring the behavioral similarities between subcastes and suggesting similarity in the neural substrates that regulate task performance in *P. morrisi* minor and major workers.

### Brain size/body size relationships differ among mature workers

**Figure 6:**
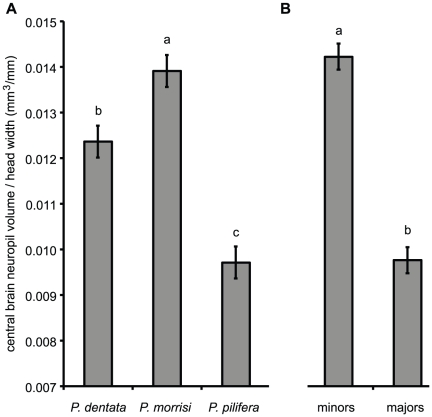
Brain-to-body-size scaling in *Pheidole* workers. Least-square mean (± s. e. m.) relative brain volumes from ANOVA model (mature workers only) indicating significant (**A**) species and (**B**) subcaste effects. Levels not sharing the same letter are significantly different ([**A**]: Tukey-Kramer *post hoc* comparisons of least-square means; [**B**]: main effect of subcaste).

Among mature workers, which we assume have completed most or all neural development, brain volume relative to body size (estimated as 2×half central brain volume/head width) differed significantly among worker groups (Figure 6; ANOVA: *F*_5,54_ = 39.3, *p*<0.0001). There were significant main effects of species identity (*F*_2,54_ = 36.9, *p*<0.0001) and subcaste (*F*_1,54_ = 121, *p*<0.0001), which together explained most of the variation among mature workers in relative brain size (R^2^ = 0.78). There was no significant species×subcaste interaction effect (*F*_2,54_ = 0.653, *p* = 0.5). Therefore, the variation in brain composition we describe exists in addition to differences in the brain-to-body-size scaling relationships of developmentally mature workers.

### Conclusions

Differences in brain size and divergent patterns of brain subregion scaling among *Pheidole* workers result in size-, age-, and species-specific patterns of investment in functional neuropil regions, reflecting the preeminent colony-wide division of labor and interspecific sociobiological variation characteristic of this species-rich genus. We believe our results have significant implications for understanding brain evolution in *Pheidole*, and in ants in general.

First, patterns of division of labor in *Pheidole* are associated with brain composition, particularly the relative sizes of olfactory input (AL) and sensory processing (MB) regions. Outside-nest work and increased behavioral flexibility correlate with greater investment in these regions. This is evident from comparisons of behaviorally differentiated workers of different ages and different subcastes, suggesting olfaction is a key determinant of task performance in *Pheidole*. Variation in subcaste brain structure is not simply due to uniform scaling of total brain size: major brains display increased relative investment in the SEG versus the central brain, but decreased investment in the ALs and MBs relative to the remainder of the central brain, likely affecting their patterns of task attendance. Interspecific variation in brain organization, which we document in addition to age- and subcaste-related effects, could explain the puzzling result that *Pheidole* major worker behavioral specialization does not appear to be correlated with their external morphology or degree of morphological specialization relative to conspecific minors [32]. While we recognize our volumetric analysis of brain subregions does not reveal details of the neuronal circuits that comprise brain neuropils, the volumetric differences we identify likely reflect fine-scale developmental differences in processes such as dendritic arborization [46] and formation of synaptic complexes (e.g. MB-C microglomeruli, [56], [57]), which presumably have more proximate impacts on the functions of behaviorally relevant neural circuits [58]. Differences in worker neural phenotype are thus relevant to understanding species-, subcaste-, and age-related division of labor. Events that occur during pupal development may produce the species- and subcaste-specific neural phenotypes evident even in the newly eclosed workers in our study, while processes that continue into the adult stage, and integrate both adaptive developmental trajectories and plastic responses to task experience, likely underlie behavioral maturation. Overall, our results support the hypothesis that division of labor in *Pheidole*, as well as in ants and other social insects, is mediated largely by neurobiological and associated upstream physiological and genetic factors [59], [60].

Second, *Pheidole* colonies, like those of most social insects, have decentralized decision-making systems [61]. Recent work has generally accentuated the “complexity” of emergent group behaviors while emphasizing “simplicity” at the individual level [62]–[65]. However, we have shown that brain structure, which underlies the ability of *Pheidole* workers to perceive social signals and cues and thus organize and participate in collective decisions and actions, varies dramatically in ways that likely affect how individuals of different subcastes and ages perceive, integrate and respond to sensory stimuli cueing behavior. Distinguishing between individual and group “complexity” may therefore be less important than understanding social and ecological factors acting on worker phenotypes, including neurobiological mechanisms regulating how workers detect stimuli and interact to produce emergent behavior. Swarm intelligence need not result simply from decision-making by large numbers of behaviorally and neuroanatomically similar individuals. The efficiency of group actions, both in insect societies [66] and vertebrate social groups [67], may well be enhanced by the presence of behaviorally sophisticated individuals whose differential task and sensory abilities can alter global patterns.

## Supporting Information

Supporting Information S1 Detailed statistical methods.
(DOC)

Table S1 Full details of ANCOVA models used to assess between-group grade shifts in brain subregion scaling for brain structures that share common scaling slopes among worker groups.
(DOC)

Table S2 Full details of ANOVA models used to assess differences among groups in absolute OL, AL, and MB volume.
(DOC)
