# Supplementary material for: Division of Labor in the Hyperdiverse Ant Genus Pheidole Is Associated with Distinct Subcaste- and Age-Related Patterns of Worker Brain Organization
Source: PLoS One. 2012 Feb 17;7(2):e31618. doi: 10.1371/journal.pone.0031618 (PMC3281964; doi:10.1371/journal.pone.0031618)
Supplement: Supporting Information S1 — Detailed statistical methods. (DOC) [file pone.0031618.s001.doc]

**Division of labor in the hyperdiverse ant genus *Pheidole* is associated with distinct subcaste- and age-related patterns of worker brain organization**

**Mario L. Muscedere & James F. A. Traniello**

# SUPPLEMENTARY MATERIALS AND METHODS

***Detailed statistical analyses***

Multivariate discriminant analyses were conducted in SPSS v. 16 among all 12 worker groups, of our three representative *Pheidole* species (sample was split into 4 subsets by age and subcaste; brains within each subset therefore differed only by species identity), and among subcastes and species in the young and mature worker cohorts (sample was split into 2 subsets by age). All size and shape variables were included in these analyses. Because workers differed substantially in body size (from a head width of 0.53 mm in a *P. dentata* minor to 1.74 mm in a *P. pilifera* major) we corrected for size variation by using the proportional volume of each subregion to the volume of the rest of the brain (either the remainder of central brain volume [ROCBV, calculated separately for each brain region] for central brain neuropils, or the entire central brain volume [CBV] for the subesophageal ganglion), or the ratio of each linear measurement to the maximal cross-sectional width of the brain across the protocerebrum. Press’s *Q* statistic (*Q* = [*n* – *cg*]2/[*n*[*g*-1]], where *n* = total number of samples, *c* = number of correctly classified samples, and *g* = number of *a priori* groups) was calculated by hand for complete and jackknifed models and compared to the *χ*^2^ distribution with 1 d.f. to determine whether classifications rates exceeded chance levels.

Bivariate scaling relationships were analysed using allometric line fitting [1] of the form y = γx^ß^, or ln(y) = ln(γ) + βln(x), where y = brain subregion volume, x = brain size (ROCBV for central brain compartments, CBV for analysis of the subesophageal ganglion), ln(γ) = α = the scaling coefficient, and β = the scaling exponent. On a log-log plot, β is the slope of the scaling relationship between y and x, and α is the y-intercept. Traditional ordinary least-squares (OLS) regression systematically underestimates regression slopes (β) when there is error in the measurement of the independent variable (as is the case in most studies of biological scaling, including ours), thus model II regression techniques such as standardized major axis (SMA) regression are preferred for scaling studies [1,2]. We therefore analysed scaling relationships between brain subregions using (S)MATR v. 2.0 [1], which fits SMA regressions to scaling data and provides a set of tools to analyse scaling differences among groups in a model II analogue of OLS ANCOVA. For each scaling relationship between subregion size and brain size we first tested whether a common SMA slope (β) could be fit to all 12 worker groups (similar to testing for homogeneity of slopes prior to an ANCOVA analysis). When (S)MATR indicated significant heterogeneity of β for a scaling relationship among worker groups, we could not further assess whether there were differences among groups in elevation or position because there was no common scaling axis (this was true for the optic lobes and central body). When a common slope was not rejected for a scaling relationship (*p* > 0.05), we used (S)MATR to test whether this slope deviated significantly from isometry (β = 1.0) using chi-squared tests, and whether there were significant shifts among worker groups in scaling elevation (differences in α, or grade shifts) or in position along the x-axis using Wald tests. (S)MATR can perform these omnibus tests, but cannot further assess whether these shifts are due to between-groups effects (i.e. effects of species, age, subcaste, or interactions among these); only systematic pairwise comparisons between all worker groups can be made. Therefore, whenever we detected significant grade shifts with (S)MATR, we performed traditional OLS factorial ANCOVAs in JMP v. 7 with species, age, and subcaste (and interactions) as fixed factors, the brain subregion of interest as the dependent variable, and brain size as the covariate. Non-significant terms were removed sequentially and models rerun until all included factors and interactions were significant; these terms indicate significant between-groups grade shifts in scaling. Slopes from these analyses are underestimates and were not reported. However, this should not strongly bias significance tests of factor main effects and interactions [3].

To test for differences among worker groups in absolute volumes of the optic lobes, antennal lobes, and mushroom bodies, and for differences in relative brain size among old worker groups, we performed factorial ANOVAs as just described for our ANCOVA analyses except we did not ln-transform structure volumes or include total brain size as a covariate. Subcaste × age interactions were significant in the analyses of optic lobe, antennal lobe, and mushroom body absolute volumes. We therefore reported differences among age cohorts separately for each subcaste as contrasts of least-square means, using the “test slices” function of JMP v. 7*.*

**SUPPLEMENTARY REFERENCES**

1. Warton DI, Wright IJ, Falster DS, Westoby M (2006) Bivariate line-fitting methods for allometry. Biol Rev 81: 259-291.

2. Ott SR, Rogers SM (2010) Gregarious desert locusts have substantially larger brains with altered proportions compared with the solitarious phase. Proc R Soc Lond B 277: 3087-3096.

3. Quinn GP, Keough MJ (2002) Ch. 12. Analyses of covariance. In: Experimental design and data analysis for biologists. Cambridge: Cambridge University Press. pp. 339-358.
