# Supplementary material for: Division of Labor in the Hyperdiverse Ant Genus Pheidole Is Associated with Distinct Subcaste- and Age-Related Patterns of Worker Brain Organization
Source: PLoS One. 2012 Feb 17;7(2):e31618. doi: 10.1371/journal.pone.0031618 (PMC3281964; doi:10.1371/journal.pone.0031618)
Supplement: Table S1 — Full details of ANCOVA models used to assess between-group grade shifts in brain subregion scaling for brain structures that share common scaling slopes among worker groups. (DOC) [file pone.0031618.s002.doc]

Table S1. Full details of ANCOVA models used to assess between-group grade shifts in brain subregion scaling for brain structures that share common scaling slopes among worker groups. CBV, central brain volume; ROCBV, remainder-of-central-brain volume.

| brain subregion | model terms | d.f. | *F* | *p* | R^2^ |
| --- | --- | --- | --- | --- | --- |
| ln(SEG volume) | *full model* | 7 | 322 | *<0.0001* | 0.95 |
|  | species | 2 | 35.6 | *<0.0001* |  |
|  | age | 1 | 6.25 | *<0.0139* |  |
|  | subcaste | 1 | 272 | *<0.0001* |  |
|  | species × subcaste | 2 | 4.92 | *<0.0089* |  |
|  | ln(CBV) | 1 | 360 | *<0.0001* |  |
|  | error | 112 |  |  |  |
| ln(AL volume) | *full model* | 7 | 131 | *<0.0001* | 0.89 |
|  | species | 2 | 8.75 | *<0.0003* |  |
|  | age | 1 | 43.6 | *<0.0001* |  |
|  | subcaste | 1 | 43.3 | *<0.0001* |  |
|  | species × subcaste | 2 | 9.17 | *<0.0002* |  |
|  | ln(ROCBV) | 1 | 387 | *<0.0001* |  |
|  | error | 112 |  |  |  |
| ln(MB volume) | *full model* | 5 | 272 | *<0.0001* | 0.92 |
|  | species | 2 | 5.71 | *<0.0043* |  |
|  | age | 1 | 296 | *<0.0001* |  |
| ln(MB volume) con’t | subcaste | 1 | 136 | *<0.0001* |  |
|  | ln(ROCBV) | 1 | 545 | *<0.0001* |  |
|  | error | 114 |  |  |  |
| ln(MB-C volume) | *full model* | 7 | 145 | *<0.0001* | 0.90 |
|  | species | 2 | 0.523 | *<*0.5943 |  |
|  | age | 1 | 43.9 | *<0.0001* |  |
|  | subcaste | 1 | 134 | *<0.0001* |  |
|  | species × age | 2 | 3.82 | *<0.0247* |  |
|  | ln(ROCBV) | 1 | 519 | *<0.0001* |  |
|  | error | 112 |  |  |  |
| ln(MB-PL volume) | *full model* | 9 | 189 | *<0.0001* | 0.94 |
|  | species | 2 | 18.3 | *<0.0001* |  |
|  | age | 1 | 500 | *<0.0001* |  |
|  | subcaste | 1 | 50.0 | *<0.0001* |  |
|  | species × age | 2 | 4.87 | *<0.0094* |  |
|  | species × subcaste | 2 | 3.66 | *<0.0289* |  |
|  | ln(ROCBV) | 1 | 420 | *<0.0001* |  |
|  | error | 110 |  |  |  |
