# Supplementary material for: Division of Labor in the Hyperdiverse Ant Genus Pheidole Is Associated with Distinct Subcaste- and Age-Related Patterns of Worker Brain Organization
Source: PLoS One. 2012 Feb 17;7(2):e31618. doi: 10.1371/journal.pone.0031618 (PMC3281964; doi:10.1371/journal.pone.0031618)
Supplement: Table S2 — Full details of ANOVA models used to assess differences among groups in absolute OL, AL, and MB volume. (DOC) [file pone.0031618.s003.doc]

Table S2. Full details of ANOVA models used to assess differences among groups in absolute OL, AL, and MB volume.

| brain subregion | model terms | d.f. | *F* | *p* | R^2^ |
| --- | --- | --- | --- | --- | --- |
| OL volume | *full model* | 7 | 40.6 | *<0.0001* | 0.72 |
|  | species | 2 | 37.7 | *<0.0001* |  |
|  | age | 1 | 0.021 | *<*0.8838 |  |
|  | subcaste | 1 | 189 | *<0.0001* |  |
|  | age × subcaste | 1 | 6.83 | *<0.0102* |  |
|  | species × subcaste | 2 | 6.62 | *<0.0019* |  |
|  | error | 112 |  |  |  |
| AL volume | *full model* | 7 | 25.9 | *<0.0001* | 0.62 |
|  | species | 2 | 28.9 | *<0.0001* |  |
|  | age | 1 | 62.4 | *<0.0001* |  |
|  | subcaste | 1 | 26.8 | *<0.0001* |  |
|  | age × subcaste | 1 | 20.3 | *<0.0001* |  |
|  | species × subcaste | 2 | 7.12 | *<0.0012* |  |
|  | error | 112 |  |  |  |
| MB volume | *full model* | 5 | 38.5 | *<0.0001* | 0.63 |
|  | species | 2 | 30.4 | *<0.0001* |  |
|  | age | 1 | 97.0 | *<0.0001* |  |
|  | subcaste | 1 | 15.5 | *<0.0001* |  |
| MB volume con’t | age × subcaste | 1 | 19.3 | *<0.0001* |  |
|  | error | 114 |  |  |  |
